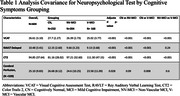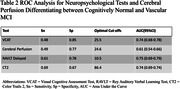# Usefulness of the Visual Cognitive Assessment Test in Detecting Vascular MCI in a Southeast Asian Cohort

**DOI:** 10.1002/alz.092329

**Published:** 2025-01-03

**Authors:** Pricilia Tanoto, Hannah En Ye, Ashwati Vipin, Yi Jin Leow, Faith Phemie Hui En Lee, Smriti Ghildiyal, Shan Yao Liew, Isabelle Yu Zhen Tan, Wayne Freeman Chong, Eunice Limin Seah, Mohammed Adnan Azam, Gurveen Kaur Sandhu, Fatin Zahra Zailan, Farid Tan Bin Hasyim Tan, Nagaendran Kandiah

**Affiliations:** ^1^ Lee Kong Chian School of Medicine, Nanyang Technological University, Singapore Singapore; ^2^ The University of Adelaide, Adelaide Australia

## Abstract

**Background:**

Visual Cognitive Assessment Test(VCAT) is a visual‐based cognitive evaluation tool which can be administered to multilingual populations without translation. VCAT has shown to be effective in differentiating mild cognitive impairment(MCI) from cognitively normal and useful for diagnosing MCI and mild AD(Kandiah et al., 2016). However, whether VCAT is also useful for differentiating between vascular and non‐vascular MCI remains to be explored.

**Method:**

604 non‐demented participants were recruited from the community as part of the Southeast Asian Biomarker and Cognition Study, Singapore(BIOCIS). All participants completed a comprehensive neuropsychological battery, including VCAT, Rey Auditory Verbal Learning(RAVLT) Delayed, and Color Trails 2(CT2). Classification of MCI was based on Petersen’s criteria(Petersen et al., 2001). Participants also underwent neuroimaging using a 3T MRI scanner. T1‐weighted and fluid‐attenuated inversion recovery MRI was used for white matter hyperintensity visual ratings as per the modified Fazekas scale (Vipin et al., 2021). Participants with Fazekas total score of 5 and above were classified as the vascular group. Therefore, participants were divided into three groups: Cognitively Normal without cerebrovascular disease(NV‐CN), Non‐Vascular MCI(NV‐MCI), and Vascular MCI(V‐MCI) and their tests mean scores and cerebral perfusion were compared. Additionally, all neuropsychological tests and perfusion were compared in differentiating between NV‐CN and V‐MCI.

**Results:**

Participant demographics included a mean age of 60.34 years±10.76, Male 44%, and mean education years of 14.44 years±3.71. After adjusting for age and education years, mean of neuropsychological test, perfusion scores were significantly different between the three groups(p < 0.05). Tukey HSD post‐hoc test showed pairwise differences between all groups, except for perfusion scores(Table 1). ROC analysis was conducted to explore the discriminative performance of VCAT in differentiating between CN and V‐MCI. The analysis indicated an AUC of 0.74 with the optimal VCAT cut‐off for differentiating between CN and V‐MCI as 25.5 with specificity 0.85 and sensitivity 0.48. In an additional ROC analysis, we compared the discriminative properties of VCAT, RAVLT Delayed, CT2 and Perfusion.

**Conclusion:**

The results of our study suggest that VCAT is useful in discriminating between CN and V‐MCI, especially in comparison to other neuropsychological tests and perfusion. Future longitudinal studies are needed for further investigation.